# Supplementary material for: Comparative genomics provides new insights into the diversity, physiology, and sexuality of the only industrially exploited tremellomycete: Phaffia rhodozyma
Source: BMC Genomics. 2016 Nov 9;17:901. doi: 10.1186/s12864-016-3244-7 (PMC5103461; doi:10.1186/s12864-016-3244-7)
Supplement: Additional file 6: — List of orphan genes with links to PFAM (related to Additional file 1: Table S1). (ZIP 1428 kb) [file 12864_2016_3244_MOESM6_ESM.zip › PFAM_UNIQUE/G04472_P.html]

#                                                                            --- full sequence --- -------------- this domain -------------   hmm coord   ali coord   env coord
  
# target name        accession   tlen query name           accession   qlen   E-value  score  bias   #  of  c-Evalue  i-Evalue  score  bias  from    to  from    to  from    to  acc description of target
  
#------------------- ---------- ----- -------------------- ---------- ----- --------- ------ ----- --- --- --------- --------- ------ ----- ----- ----- ----- ----- ----- ----- ---- ---------------------
  
G04472\_P             -            546 Cytochrome-c551      PF10643.4    233     0.038   11.9   0.2   1   1   1.4e-05     0.082   10.9   0.2   109   190   163   241   158   263 0.77 - Photosystem P840 reaction-centre cytochrome c-551  
G04472\_P             -            546 MAD                  PF05557.8    722       5.2    3.7  48.3   1   2     0.024       5.7    3.5   4.0   222   267     6    49     2    60 0.86 - Mitotic checkpoint protein  
G04472\_P             -            546 MAD                  PF05557.8    722       5.2    3.7  48.3   2   2    0.0069       1.7    5.3  36.9    48   265    59   304    50   350 0.72 - Mitotic checkpoint protein  
G04472\_P             -            546 Mononeg\_RNA\_pol      PF00946.14  1072      0.77    5.7   8.6   1   1   0.00017         1    5.3   8.6     1   151    87   233    87   325 0.75 - Mononegavirales RNA dependent RNA polymerase  
G04472\_P             -            546 PYRIN                PF02758.11    83      0.12   10.8   2.3   1   2   4.7e-05      0.28    9.6   0.1    20    59    23    65    21    74 0.87 - PAAD/DAPIN/Pyrin domain  
G04472\_P             -            546 PYRIN                PF02758.11    83      0.12   10.8   2.3   2   2     0.071   4.2e+02   -0.6   0.2    34    62   281   310   273   328 0.69 - PAAD/DAPIN/Pyrin domain  
G04472\_P             -            546 T2SE                 PF00437.15   272      0.14    9.7   1.7   1   1    0.0031      0.33    8.5   1.7    18    83   190   256   175   306 0.76 - Type II/IV secretion system protein  
G04472\_P             -            546 TcdB\_N               PF12918.2     66      0.45    9.3   3.5   1   3   0.00011      0.64    8.8   0.6    16    45   164   193   159   196 0.87 - TcdB toxin N-terminal helical domain  
G04472\_P             -            546 TcdB\_N               PF12918.2     66      0.45    9.3   3.5   2   3      0.31   1.9e+03   -2.3   0.1    21    37   213   227   203   238 0.67 - TcdB toxin N-terminal helical domain  
G04472\_P             -            546 TcdB\_N               PF12918.2     66      0.45    9.3   3.5   3   3      0.48   2.9e+03   -2.9   0.0     4    21   228   244   225   246 0.78 - TcdB toxin N-terminal helical domain
